# Looking back at a life in sleep research—and some thoughts for the future

**DOI:** 10.1093/sleepadvances/zpac031

**Published:** 2022-09-12

**Authors:** Wallace B Mendelson

**Affiliations:** Department of Psychiatry, The University of Chicago (ret)

**Keywords:** neuropharmacology, pharmacology—sedative-hypnotics, pharmacology—wake promoting agents, REM sleep, melatonin, endocrinology, psychiatry, sleep deprivation

## Abstract

In reviewing my studies, some of which are nearing the half century mark, I’ve described work on sleep-related growth hormone, the effects of hypnotics on the perception of sleep, REM sleep induction in humans by cholinergic drugs, the benzodiazepine receptor, the anatomic sites of action of hypnotics, the endocannabinoid system and sleep, and the relation of anesthesia to sleep. Special mention along the way goes to cases in which drugs produced totally unexpected effects, including methysergide producing opposite effects on growth hormone secretion in sleep and a waking provocative test, the converse actions on sleep of the B-10 benzodiazepine enantiomers, and the promotion of wakefulness by microinjection of the hypnotic triazolam into the dorsal raphe nuclei. This work is placed in the context of what was known at the time, as well as what has been observed in subsequent years. Many of these studies indicate that the medial preoptic area may be a common site for the sleep-promoting action of a wide range of agents including traditional hypnotics, ethanol, propofol and melatonin. In the future it may be worthwhile looking at the beta-carbolines, and also the endocannabinoid system, when exploring drugs with new mechanisms of action for treating sleep/wake disorders. An Addendum to this paper describes memories of working with Frederick Snyder, J. Christian Gillin, Richard Jed Wyatt, and Floyd E. Bloom.

**Statement of Significance**

This is a description of one investigator’s pathway through several decades of sleep research-- how he and the field both evolved, and the particular fascination of unexpected observations.

## Introduction

The early 1970s were heady times for a young psychiatry resident with an interest in sleep. In 1972, Michel Jouvet, building on the results of his pioneering studies of brainstem lesions in animals, proposed a model of sleep regulation involving the interaction of serotonergic neurons in the dorsal raphe and noradrenergic neurons of the locus coeruleus in the genesis of NREM and REM sleep [1]. Subsequently, a number of studies were designed to explore its implications and to track the time course of changes in sleep following alteration of function of these neurotransmitters [2]. At that time, the biogenic amine hypothesis of mood disorders, derived from studies of reserpine, MAO inhibitors and other drugs, and suggesting that a deficiency of serotoninergic or noradrenergic activity was at the root of mood disorders, continued to gain support. Similarly, the growing recognition that some hallucinogens such as mescaline and LSD had structural similarities to catecholamines and indolamines had for some time supported a parallel theory involving disturbed monoamine function in psychoses [3]. Interestingly, the drugs used to explore all three of these hypotheses about monoamines were similar, including precursors, metabolism inhibitors, and receptor blockers.

In 1971, the exigencies of the Vietnam years placed me at St. Elizabeths Hospital in Washington, DC, and the kindness of Floyd Bloom (more about this later), led me to the laboratory of Richard Jed Wyatt, where many of these studies were going on. St. Elizabeths, which first opened in 1855 as the Government Hospital for the Insane, and which had played a role in caring for Union soldiers during the Civil War, was by the 1970s a large campus-like NIMH facility for chronic patients, with a forensic unit known for its ‘White House cases’ (persons who had threatened the President), and a research arm in the William Alanson White Building.

Richard Wyatt was a remarkable psychiatrist intent on exploring the regulation of sleep as well as the biological underpinnings of schizophrenia. Like Jouvet and others, he built his work on the new knowledge of monoamines which was coming to light, based on studies in the 1960s of infusions into young birds (who did not have a well-developed blood–brain barrier), the elaboration of nervous system pathways made possible by tissue fluorescence techniques [4], and lesions of the newly discovered pathways in animals. When I first met him, he had just published a widely-read paper entitled ‘The serotonin-catecholamine dream bicycle: a clinical study’ [5] which postulated that at a human level an alternating dominance of the two groups of transmitters, much as the rotation of bicycle pedals, was responsible for the NREM–REM cycle.

Richard did not have an active animal sleep laboratory at that time. He personally taught me the surgical techniques and how to read the sleep records, and set me to work on studies of REM deprivation. We used the quaint method of placing rats on inverted flower pots surrounded by water, so that when REM sleep appeared the resulting muscle atonia led them to fall into the water and wake up. This technique had been employed by others, but characteristically Richard had me first validate it with 24-h recordings before going on to studies of its effects on intermediary metabolism. At the same time, as part of my Public Health Service assignment, I was involved in the medical aspects of the human sleep program, and learned the techniques of human sleep studies.

As it happened, I began to work with one of the young investigators, Chris Gillin, who was 2 or 3 years ahead of me in his training, but like me was fulfilling his military obligation. During this time, we became familiar with the drugs used to modify levels of biogenic amines, such as 5-HTP, PCPA, AMPT as well as receptor agonists and antagonists. I emphasize this, because in some ways it became a natural step to use pharmacologic tools in dealing with topics we later became interested in. In my case, they were applied, for instance, to understanding sleep-related growth hormone secretion, paradoxical insomnia, and the location of action of hypnotics in the CNS. Chris was to apply them to the understanding of the cholinergic system in REM initiation in normal volunteers and depressed patients, and of course in many other areas. This theme will reappear a number of times as we look at various areas of study in roughly chronologic order.

### Sleep-related growth hormone (GH) secretion

My 2 years at St. Elizabeths completed, I returned to Washington University to finish my residency and go on staff. There I was approached by the endocrine group to do further studies following their discovery of sleep-related GH secretion [6]. They had observed that in young adults there is a rise in plasma GH concentrations during the first 90 min of sleep, lasting 1.5–3.5 h and generally occurring in slow-wave sleep (stage N3). If sleep were delayed, so was the secretion of GH. If subjects were awakened and stayed up for 2–3 h, a new secretary episode would occur after they returned to sleep. As one of the first studies in my own lab, I applied the lessons from St. Elizabeths to look at which neurotransmitters might be involved in sleep-related GH secretion. The serotonergic system seemed a good place to start, as 5-HTP, which I had had experience with there, was known to increase daytime resting concentrations of GH [7].

**Figure 1.**
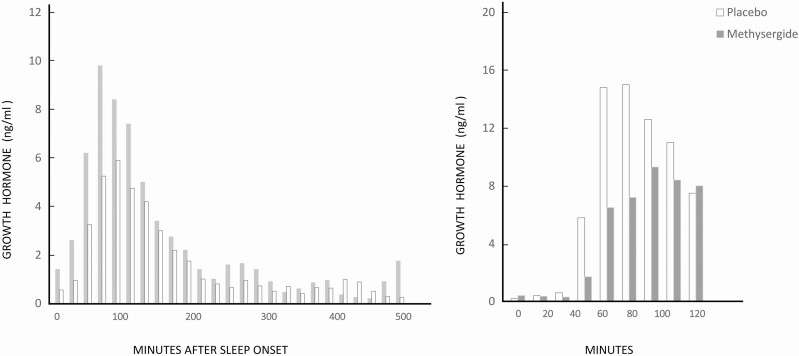
Effects of methysergide (gray bars) and placebo (white bars) on sleep-related GH secretion (left), and on GH secretion during a daytime insulin tolerance test (right). Adapted from reference [8].

The first probe I chose was methysergide, an ergot alkaloid then known as a serotonin antagonist (in subsequent decades and the characterization of receptor sub-types its actions have turned out to be more complex) used for migraine and cluster headaches, and known to inhibit daytime insulin-stimulated GH secretion. We gave methysergide 2 mg or placebo orally to normal volunteers every 6 h for 48 h, and then performed nocturnal sleep studies and daytime insulin tolerance tests (ITT) on normal volunteers. As expected, methysergide suppressed GH secretion in response to insulin; to our surprise, however, it had the opposite effect during sleep (Figure 1), causing a substantial increase in secretion [8]. The implication seemed to be that—in the case of GH—the neurotransmitter regulatory mechanisms were quite different during a test in the waking state and during sleep.

Over the next 6 or 7 years, we gave drugs acting on different transmitters, such as dopamine, methscopolamine, and piperidine. Again, differences between effects during the waking ITT and sleep appeared: methscopolamine had a marginal inhibitory effect on GH in the ITT, but virtually abolished its secretion during sleep. Ultimately we were able to produce a diagram of the overall differences in neurotransmitter regulation of GH in the two situations [2].

With the hindsight of several decades, I realize that our conclusions from those days require clarification of circadian effects; at the time of our study, a 12 h sleep-wake reversal study had found an immediate shift of GH secretion to the new sleep time, implying little circadian involvement [9]. Eight years after our work, however, a jet lag study suggested some possible circadian role [10], as did a 2004 study of night workers in 24 h of bed rest and dim light [11]. The methysergide observation was a turning point for me, however, with the realization that drugs might have very different effects during sleep. In terms of our field, it added to the notion of a unique physiology of sleep put forth by Bill Dement and others. Most of this argument traditionally focused on REM—the lack of thermoregulation and decreased ventilatory response to hypercapnea, for instance—but the case of GH recognized differences between waking and NREM sleep.

### Induction of REM sleep in humans by cholinergic drugs

At a Society of Neuroscience meeting in St. Louis in about 1974, both Chris and Richard lured me back to NIMH. Things had changed in the few years I had been gone. Fred Synder, who had done groundbreaking work at the Bethesda campus on pharmacology of sleep, evolutionary aspects of sleep and dreaming, and many other areas, was leaving, and Chris moved to the Bethesda human sleep lab, where I joined him while still doing animal work at St. Elizabeths.

It was in 1975 that Alan Hobson and Bob McCarley [12] first published the reciprocal interaction model, positing complex cyclic activity of monoaminergic and cholinergic cells in the brainstem in the formation of NREM and REM sleep. While this drew my attention to the use of cholinergic probes in my animal studies, Chris and Natraj Sitaram were drawn to the idea that drugs which enhance cholinergic activity might alter REM sleep in humans [13]. It turned out that infusion of physostigmine (and later arecoline) 35 min after sleep onset reliably induced the appearance of REM sleep. Further work found that patients with depression, both active and in remission, were more sensitive to this effect than controls, suggesting that this might be a biological marker for a vulnerability for mood disorders [14]. I mention these studies, in which I had a minor role, because once again they showed the utility of applying pharmacologic probes to study the regulation of physiologic processes. I also bring them up here because of the remarkable impact they had on me personally: it was an amazing experience to infuse a drug and watch it bring about the appearance of REM sleep a few minutes later. It was one of the times, along with observations of drugs having absolutely unanticipated effects, that evoked a sense of wonder.

### The role of the GABAa-benzodiazepine receptor complex in sleep induction, and novel ligands such as the beta-carbolines

The benzodiazepines (BZs) burst upon the world in 1960 with the anxiolytic chlordiazepoxide (Librium), followed by diazepam (Valium) and then in 1970, flurazepam (Dalmane), the first benzodiazepine with an indication for sleep. From 1969 to 1982 they were the most widely prescribed medicines in the US (and in 1977, worldwide). Within 2 years of its release, flurazepam was the most frequently prescribed hypnotic in America. As we look back with a perspective of 60 years, and a recognition of the many serious concerns about their use—issues for instance of dependence, daytime sedation and cognitive impairment—it is difficult to picture this enthusiasm without looking at the context. At the time, the dominant hypnotics were the barbiturates and equally toxic agents such as methaqualone (Quaalude) and glutethimide (Doriden). The hypnotic barbiturates were lethal in overdose (often with as little as 10 doses), had significant respiratory depressant qualities, and stimulated the cytochrome P-450 system. At the time of introduction, BZs were thought to be less harmful in these and other ways [15]. It is a good reminder to be cautious about the benefits of new drugs until there is sufficient clinical experience.

In spite of the popularity of BZs in these early years, however, the mechanism by which they produced their pharmacologic effects was unclear for almost 2 decades. Then in 1977, two independent groups discovered that diazepam binds to high affinity, saturable sites in the nervous system, which were commonly termed the ‘benzodiazepine receptor’ [16, 17]. They were thought to be part of macromolecular complexes comprised of three functional components, including recognition sites for benzodiazepines and GABA, and a chloride ion channel. In essence, benzodiazepine receptors were thought to be allosteric modulatory sites of GABAa receptors, and as time went on they were found to have a number of sub-types modulating different pharmacological effects.

In the first years after this discovery, it became clear that the BZ receptor mediated the anxiolytic, muscle-relaxant and anticonvulsant properties of BZs, but it was not clear whether this was true of their sleep-inducing effects. Beginning in 1982, pharmacologist Phil Skolnick at the NIH, organic chemist James Cook at the University of Wisconsin, and I undertook a wide variety of studies to answer this question, explore the intracellular mechanisms by which stimulation of the receptor produces pharmacologic actions, and to study a number of interesting ligands. Among these were studies of the B-10 benzodiazepine enantiomers and the beta-carbolines.

### The B-10 enantiomers

Drug receptors are considered to have a number of properties which we have already mentioned such as high affinity and saturability, and another key element is stereospecificity. Although many clinical benzodiazepines such as diazepam and flurazepam lack asymmetrically arranged carbon atoms, there are some which have them, including what are known as the B-10 enantiomers (compounds which are non-superposable mirror images). The (+) B-10 enantiomer (RO 11-6896) has about twice the affinity of flurazepam for the benzodiazepine receptor, while the (−) B-10 (RO 11-6893) is roughly two orders of magnitude less potent [17]. Both showed increased affinities in the presence of GABA, a property typical of agonists, leading us to predict that the (+) enantiomer would be a potent sleep-inducing agent, and that the (−) would be substantially weaker.

As it turned out, we were only half right. As expected, the (+) B-10 substantially reduced sleep latency in rats, but the (−) compound, instead of just having a weaker action, had exactly the opposite effect, raising sleep latency [2, 18]. We then confirmed that these properties were mediated by the BZ receptor, as they were prevented by the BZ receptor blocker CGS 8216. The results of the B-10 studies indicated, then, that the actions of BZs on sleep were stereospecific, and mediated by the BZ receptor. At a more personal level, I was fascinated with the prospect that a drug might induce sleep, while its mirror image increased wakefulness. While I was aware that this principle had been seen before—for some barbiturates, for instance, the (+) and (−) forms have opposite effects on seizure threshold or excitability of cultured spinal neurons—it was fascinating to observe. In my less scientific moments, I was reminded of the scene in *Alice in Wonderland* in which Alice eats from the right side of a mushroom, causing her to shrink, and then tastes the left side, which causes her to grow [19].

### The beta-carbolines and sleep

Beta-carbolines comprise a family of alkaloid compounds found in plants and animals, including the mammalian brain. There are over one hundred natural and synthetic variations, the latter derived from structures bearing an indole ring, such as tryptophan, combined with a pyridine ring. Their diverse structures have led to a wide range of actions including modulation of anxiety, pain, seizure threshold and food consumption, neuroprotection and memory improvement, as well as possible anti-tumor and antimicrobial effects. They bind with varying potencies to the benzodiazepine receptor, and can act as agonists, antagonists or inverse agonists. The richness of the range of both receptor actions and resulting pharmacologic properties made them ideal as probes for Phil Skolnick, James Cook, and I to explore the possible role of the benzodiazepine receptor in sleep in the early 1980s. Here we will describe two of them, abbreviated as 3-HMC and B-CCT.

To give this some context: in the first years after the discovery of benzodiazepine receptors, it became clear that they mediated the anxiolytic, anticonvulsant and muscle-relaxant properties of BZs, but it was not yet certain whether this was true for sleep induction. In order to explore this, we administered the beta-carboline 3-HMC, which was known to block the anticonvulsant and anxiolytic effects of diazepam, to rats in a daytime sleep study. 3-HMC was found to increase sleep latency and decrease total sleep time in a dose-dependent manner, and prevented the sleep-inducing effects of flurazepam [20]. Its arousing effect was prevented by the BZ receptor blocker CGS 8216, indicating that this was indeed mediated by the binding of 3-HMC to the BZ receptor. 3-HMC, while increasing wakefulness in rats, had relatively little effect on motor activity, suggesting that its actions were relatively specific to sleep/wake mechanisms rather that to general behavioral activation.

As an additional way of confirming the relation of the BZ receptor to sleep, we turned to another agent, B-CCT, which is selective for the alpha-1 subtype, and which does not have anxiogenic or convulsant properties. Like 3-HMC, it increased sleep latency and decreased total sleep, in a time course which paralleled its occupancy of the BZ receptor, again confirming the relation between the two [2, 21]. Another implication was that B-CCT or some related compound might potentially be useful for development as a clinical analeptic. Subsequent studies have found that it reduces benzodiazepine-induced ataxia in monkeys [22] and apparent anxiety in a rodent model of benzodiazepine withdrawal [23].

Indeed, as the years have gone on, there has been continued interest in developing beta-carbolines for a variety of potential clinical uses, among these the modulation of alcohol consumption. A beta-carboline with mixed agonist-antagonist properties which selectively binds to the alpha-1 subtype, 3-PBC reduces alcohol-maintained responding in a rodent model when infused into the ventral pallidum [24]. Another beta-carboline, 3-ISOPBC·HCl, reduces behavior analogous to binge drinking without evidence of behavioral sedation in a model involving maternally-deprived rats [25]. Some beta-carbolines have also been found to have potential antidepressant properties in animal models [26]. Others have been found to have analgesic effects in mouse tail-flick tests, apparently due to kappa opioid receptor agonism [27].

### The medial preoptic area as a locus of action of many sleep-promoting drugs

**Figure 2.**
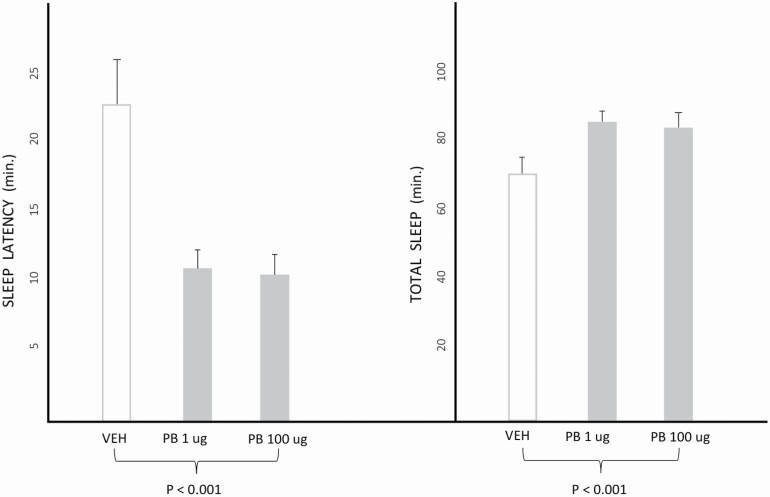
Effects of pentobarbital (PB) 1 µg and 100 µg and vehicle microinjected into the medial preoptic area of rats on sleep latency and total sleep time. The study consisted of 2-h recordings performed after microinjection at 10:00 A.M., under 12:12 h L:D conditions such that lights come on at 8:00 A.M. Adapted from reference [31].

The beta-carboline studies were designed to give some insight into how hypnotics function at a molecular level, but left open the question of where they might act anatomically. In order to pursue the question, I began a series of hypnotic microinjection studies while still at the NIMH in 1987, which continued though some of the last studies at the University of Chicago in 2001. As a starting point it seemed reasonable to look at sites which were known to affect sleep as determined by lesion or electrical stimulation studies, and on this basis we set out to examine a number of locations in the brainstem, basal forebrain and hypothalamus. We also wanted to make sure that any effects we saw were not due to diffusion into the ventricles; for that reason we determined that doses of up to 1 ug of triazolam infused into the lateral ventricle of rats did not induce sleep, and chose for our microinjection studies 0.5 µg. We then administered it into a variety of sleep-related loci during daytime sleep studies in rats, including the horizontal limb of the diagonal band of Broca, the medial and lateral preoptic areas, the dorsal raphe nuclei, and the locus coeruleus [28, 29], and later the ventrolateral preoptic area [30]. Interestingly, the only one in which sleep latency was shortened and total sleep increased was the medial preoptic area of the hypothalamus, an integrative area regulating sleep and temperature and various activities such as sexual behavior. Over the years, we found the same result from administering pentobarbital (Figure 2), ethanol, adenosine, propofol and melatonin into the same locus [29, 31–34]. With some of these, including ethanol, adenosine and propofol, we also confirmed that the sleep-inducing property was blocked by co-administration of the benzodiazepine receptor blocker flumazenil.

In summary, the results with a wide range of types of sleep-inducing agents, from classical hypnotics to a clinical anesthetic and even melatonin, suggest that the medial preoptic area might play an important role in pharmacologic sleep induction. The initially surprising finding that melatonin is among these might have been anticipated by an earlier study indicating that melatonin enhances GABA concentrations in the hypothalamus [35], and is consistent with a later study that flumazenil prevents sleep induction by parenterally administered melatonin in the rat [36]. Interestingly, a 2021 study has found that the dual orexin receptor antagonist TCS-1102 injected into the medial preoptic area (which receives input from orexin neurons) increases NREM and REM sleep in lactating rats [37]. To put these microinjection studies in a broader context, the preoptic area and the basal forebrain have come to be seen as two of the major regulatory centers promoting sleep [38]. Our reports of sleep induced by a wide range of substances added a pharmacologic arm to the understanding of the medial preoptic area, suggesting that it may be a target of their action.

Before leaving the topic of anatomical sites of action of hypnotics, I’d mention one other curious finding. Though injections of triazolam into the majority of sleep-related sites (with the very notable exception of the medial preoptic area) usually produced no effects, injections into the dorsal raphe nuclei increased sleep latency and decreased total sleep time [39]. In retrospect this seemingly paradoxical finding—that a drug which promotes sleep when given systemically would increase awakening when given locally—might have been predicted from previous studies showing behavioral activation after injection of GABA agonists into the dorsal raphe [40], and that benzodiazepines may decrease neuronal activity there [41]. It seems likely, then, that the increased wakefulness in rats after injection of triazolam into the dorsal raphe was a pharmacological counterpart to Jouvet’s studies of the results of lesions there [1]. On a more personal level, it was once again one of those moments—like seeing methysergide increase sleep-related GH secretion or B-10(−) increase wakefulness—in which a drug did something totally unexpected, and indeed opposite to my predictions.

### Effects of hypnotics on the perception of sleep in humans

In 1987 I moved to SUNY Stonybrook, and then to the Cleveland Clinic in 1993. In both cases I was managing large sleep disorder centers and fellowship programs, and this clinical immersion led me to become more engaged with the management of chronic insomnia. I had always been impressed with the seminal study by Al Rechtschaffen, in which he awakened poor sleepers 10 min after the first sleep spindle, and found that they were more likely than good sleepers to say they believed they had been awake [42]. In the 1980s, several groups including mine made similar observations in more fully characterized insomnia patients. Additionally, Bonnet and Moore [43] had carefully described the timing of the perception of being asleep in normal sleepers. They found that the auditory arousal threshold began to rise after the first sleep spindle, but it was not until 2–4 min later that at least half of the subjects believed they had been asleep. Thus the time course of two objective measures of sleep (the EEG and auditory arousal threshold) and the subjective perception of being asleep had somewhat different time courses. It seemed possible, then, that some patients might have an exaggeration of this normal difference. Though the early clinically-described forms of insomnia (including paradoxical, psychophysiological, idiopathic, and others) have now been subsumed into the blanket category of ‘chronic insomnia’ in the ICSD-3, at that time, ‘subjective’ or ‘paradoxical’ insomnia was considered a separate entity. In the current classification, it is still recognized that one aspect of insomnia is that some patients may manifest a larger than normal discrepancy between subjective and objective measures of sleep.

The pharmacological counterpart to this is that the subjective benefit of hypnotics often differs—frequently exceeding—the changes as measured on the EEG (e.g. [44]), I wondered whether part of this difference could be explained by some sort of drug-induced cognitive change. One possibility of course was that hypnotics might distort the sense of time, accounting for differing reports of amount of sleep; in early studies with flurazepam [45] and triazolam [44] this did not appear to be the case with the doses we used and the setting of our nocturnal sleep studies. In order to explore this further, we set up a protocol in which subjects were awakened by a progressive auditory stimulus at five time points across the night:

1. Five minutes after ‘lights out’.
2. 10 minutes after the first sleep spindle.
3. Five minutes after the beginning of stage 4 (N3).
4. Five minutes after the beginning of REM sleep.
5. At the onset of the first awakening after the first REM episode.

We administered triazolam in three doses (0.125, 0.25, and 0.5 mg) and placebo to chronic insomniacs and gave them a questionnaire about their sleep at each of these five time points, in a repeated measures design. On placebo, they reported having been asleep in 26.7 per cent of the forced arousals; during all possible drug conditions (3 doses and 5 time points) on triazolam this rose significantly (*p* < .0001) to 56.5 per cent [46]. More detailed questioning suggested that on drug nights, particularly at the time point five minutes after ‘lights out’, subjects were less certain about their responses of being awake or asleep, and many reported imagery changed from ‘participating’ to ‘watching’ or not watching or participating.

In order to determine whether these changes in reports of being awake or asleep were unique to triazolam, we repeated the study with 30 mg flurazepam and 10 mg zolpidem [47]. This time, on placebo insomniacs perceived themselves as having been asleep on 30.9 per cent of the forced arousals, rising to 40.4 per cent with flurazepam (NS) and 54.7 per cent with zolpidem (*p* < .03). We then repeated the study with normal volunteers [48]. This time reports of having been asleep on placebo, flurazepam, and zolpidem were 40.3, 42.9 and 47.9 per cent (NS). The overall finding, then, was that hypnotics from two different pharmacologic classes altered the perception of being awake or asleep and that this effect was specific to chronic insomniacs. This seemed to raise the possibility that these cognitive changes might help explain one aspect of the reported subjective benefits of hypnotics, and their often-observed discrepancy from objective measures. Many questions remain, including whether these drug-induced changes in perception, which were primarily oriented to the first NREM–REM cycle, appear in later cycles, and whether this effect persists in chronic administration.

### Oleamide

The family of unsaturated fatty acids have long been thought to have a role in modulating receptor ion channel function in the nervous system [49]. Among these is the fatty acid amide oleamide, an endogenous substance indirectly affecting cannabinoid receptors, formed in microsomes in the CNS, and found in human plasma. Oleamide does not directly bind to cannabinoid receptors; its effects may be modulated by increasing concentrations of the endogenous CB-1 receptor agonist anandamide (also a fatty acid amide) by interfering with metabolism or uptake. While it does not alter binding of ligands to GABA or serotonin receptors, it enhances currents gated by 5-HT2a, 5-HT2c and GABAa receptors.

**Figure 3.**
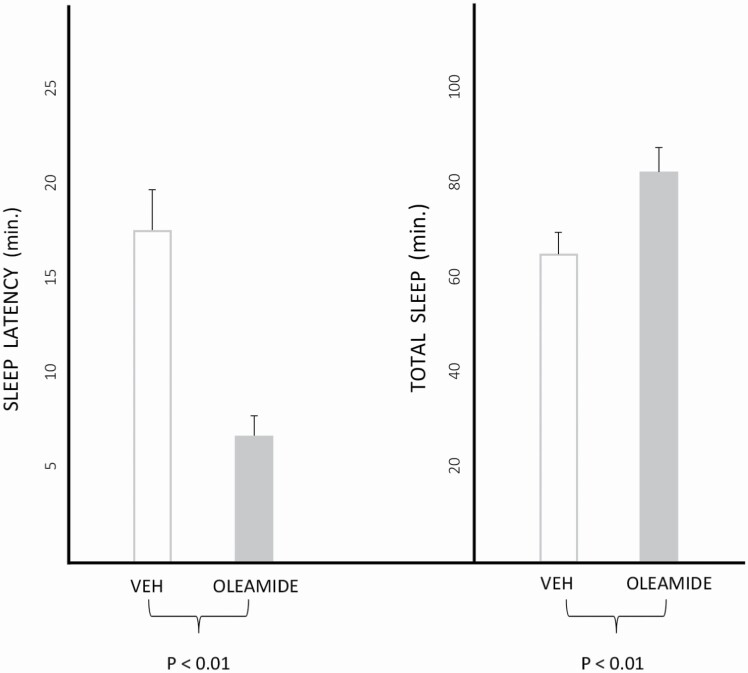
Effects of oleamide 10 mg/kg and vehicle administered intraperitoneally to mice on sleep latency and total sleep time. The study consisted of 2-h recordings performed after injection at 10:00 A.M., under 12:12 h L:D conditions such that lights come on at 8:00 A.M. Adapted from reference [49].

There had been a longstanding association of oleamide and sleep since the mid-1990s, when it had been detected in the CSF of sleep-deprived cats [50], and observed to decrease motor activity and induce what appeared to be behavioral sleep in rats [51]. A few years later, I collaborated with Anthony Basile of the NIH in characterizing this relationship further. After preliminary studies in which we confirmed that peripherally administered oleamide in rats decreased motor activity, and that endogenous oleamide rose by 300–400 per cent after sleep deprivation for 6 h, we found that 2.8 and 5.6 µg given intra-ventricularly in the daytime shortened polygraphically measured sleep latency to 44–64 per cent of control measures [52]. In a follow-up study, we observed that 10 mg/kg given intraperitoneally shortened sleep latency, while increasing NREM and total sleep time (Figure 3) in mice [49]. An inhibitor of oleamide metabolism (LOH-1-1-151) was found to shorten sleep latency and increase total sleep time when given intra-ventricularly to rats, and intra-peritoneally to mice [53]. A CB-1 receptor blocker, SR-141716, in doses which had no effect on sleep when given by itself, prevented sleep induction by oleamide in rats [49]. When very low doses of intra-ventricular oleamide and triazolam, which did not affect sleep by themselves, were given together, the result was a potent hypnotic action [54]. We also found that in mice lacking the beta-3 subunit of the GABAa receptor there was a limited hypnotic effect of oleamide [55].

In summary, oleamide is an endogenous substance which accumulates in the mammalian CSF after sleep deprivation. Since its hypnotic effect is prevented by a CB-1 antagonist, though it does not itself bind there, it seems likely that it is mediated by a presumably indirect action on cannabinoid receptors. Its synergism with triazolam opens the possibility that the GABA system is involved as well, either directly or by a downstream pathway. Among the implications of these studies is that oleamide might join the already burgeoning list of possible endogenous sleep regulating substances, for which it meets some of the criteria [56]. Moreover, it seems reasonable that oleamide, or some related compound, might potentially be developed for clinical use as a hypnotic.

### Sleep and anesthesia

In 1996 I moved to the Sleep Research Laboratory at the University of Chicago, after Al Rechtschaffen retired. One of the major projects there was the oleamide studies. Another took advantage of two coincidences—my growing familiarity with Al’s disk-over-water sleep deprivation technique, which I was using in aging studies, and a visit from Avery Tung, a faculty anesthesiologist. Avery noted that propofol had made it possible to keep patients sedated for prolonged periods in the ICU, and wondered whether the occasional episodes of hallucinations and other symptoms when sedation stopped might be due to sleep deprivation having accumulated during sedation. In a broader sense, he was asking about the relationship of anesthesia to homeostatic regulation of sleep. In order to explore this, we sedated rats with intravenous propofol for 12 h which included their normal sleep period, and then compared the EEG pre- and post-treatment [57]. If they had been sleep-deprived during sedation, we would have expected the transient elevation in the NREM delta power band, and increases in NREM and REM sleep, which Al had so well characterized. In fact, none of these things happened. The implication seemed to be that during prolonged sedation the rats had not accumulated a sleep debt.

In a follow-up study, we used the disk-over-water apparatus to sleep deprive rats for 24 h; afterwards, half were given propofol for 6 h, while controls were allowed to sleep ad libitum [58]. During this period, both groups were given intravenous lipid nourishment. Our reasoning was that if propofol sedation did not allow for recovery from sleep deprivation, then the recovery process—marked by elevations in NREM delta power, as well as amounts of NREM and REM sleep—would be delayed until the end of the 6 h drug treatment. As it turned out, the timing of recovery, lasting about 12 h, was indeed the same in both groups. This seemed to suggest that the recovery process from sleep deprivation was also able to occur during the propofol sedation. As mentioned earlier, we had also shown that propofol injections into the medial preoptic area induced sleep in a manner similar to a number of traditional hypnotics, and was prevented by flumazenil. Cumulatively, these studies seemed to suggest an overlap of regulatory mechanisms of sleep and anesthesia.

### Unfinished business

The questions never stop coming. As the last section indicates, it would be fascinating to pursue the relationship of anesthesia and sleep and explore their commonalities as well as their differences. Many other areas, and questions, come to mind. Here are a few.

### Drug effects on sleep perception

Certainly one question which comes up is whether tolerance develops for hypnotic-induced alterations in sleep perception during chronic use. Would drugs which work by newer mechanisms of action, such as orexin antagonists, alter perception of sleep in the same way that triazolam and zolpidem do?

### The beta-carbolines

This is a rich class of compounds which continue to be explored for a variety of uses. We have previously mentioned animal studies suggesting that they might be beneficial in modulating alcohol consumption or as antidepressants. They have also been proposed as tools for studying Alzheimer’s disease [59], and have been used in a promising human trial as a treatment for malaria [60]. It seems possible that some of these versatile compounds might be explored for promoting wakefulness, as we did with B-CCT. This also points to a broader principle: although there has been much attention to receptor blockers in various contexts (e.g. suvorexant), it may be a useful strategy to be on the lookout for inverse agonists for receptors of interest, which may potentially have useful properties.

### Oleamide studies

In our rat studies, oleamide had potent hypnotic effects, an observation supported by subsequent work (e.g. [61], Mice lacking the enzyme FAAH, which metabolizes oleamide and anandamide, have been found to have increased NREM sleep [62]. As the years have gone by since our work, many have proposed that the family of fatty acid amides might provide the basis for future drugs, imaging agents, and research tools [63]. A recent review emphasizes the observations that oleamide, anandamide, and 2-arachidonylglycerol promote NREM and/or REM sleep due to actions involving the CB1 receptor, suggesting that the endocannabinoid system might be a target for developing new treatments for sleep disorders [64]. This is an intriguing approach for potentially finding hypnotic agents with a new mechanism of action.

## Addendum: Memories of Fred Synder, Chris Gillin, Richard Wyatt, and Floyd Bloom

Below are a few comments about these remarkable persons whom I have been lucky to know. This section in no sense represents their biographies, and I have made no attempt to list all their achievements. Instead, this represents personal memories of how our lives intersected.

## Frederick Snyder MD

Fred Snyder was the director of the sleep laboratory at the Intramural Program of the NIMH before Chris Gillin and myself. When coming back to the NIMH in 1975 I was already familiar with his pharmacologic papers in the early 1970s, with Richard Wyatt and often in collaboration with David Kupfer and others, on the effects of L-DOPA, MAO inhibitors and l-tryptophan on sleep, and with Chris Gillin on glucocorticoids and sleep. The study with MAO inhibitors included one of the early statements of the idea that REM deprivation (in this case pharmacologically induced) might have antidepressant properties. He was particularly interested in the phylogeny of sleep, traveled widely to study animals in their natural habitats, and maintained a lab at the National Zoo, where EEG telemetry signals from large animals were transmitted to antennas buried in the cage floors. In the 1960s he proposed that REM sleep was originally a survival mechanism, onto which dreaming was grafted at a later evolutionary point. He was particularly interested in primates, as he thought studying them might help bridge the gap from small laboratory animals to humans in understanding REM and dreaming. He also suggested that in primitive societies it was advantageous for individuals to have varying chronotypes, so that some members could be alert to danger when others slept. His last study, completed by Chris in 1979 after he left, involved using multivariate analysis to characterize sleep EEG profiles of normal, insomniac and depressed subjects, recognizing them in about 75 per cent of cases.

One of my first impressions of Fred came about by chance. I had moved into a house in the Maryland suburb of Kensington, which turned out to be near where he had lived. He was well known among the neighbors for his attachment to a young primate which he kept at his home for years, and his sadness when it became too big and had to be sent to a preserve in Texas. It was said that he had spent a great deal of time and care selecting the right place, in the same way one would when finding a facility for a family member.

In about 1974 Fred moved on from academe to become a physician on an Indian reservation in the western states, also served by the PHS. These were happy years for him, but he ultimately suffered a stroke which greatly impaired him motorically. Shortly thereafter, he visited the NIMH lab, and he and Chris and I spent one of the most stimulating days in memory talking about sleep research. Later, unable to continue his work on the reservation, he and his wife moved to Hawaii, where I am told he practiced and lived on the grounds of Mehelona Memorial Hospital, on Kauai. It was there that he passed away in February 1979.

## J. Christian Gillin MD

When I first met Chris in 1971, he had recently come from a psychiatry residency at Stanford, where he had worked with Bill Dement and first became involved in sleep research. He liked to tell the story of how Bill had given him a bell jar containing a drug, and asked him to inject it into rats and measure their sleep response. When the results were not at all what was expected, Bill asked him to review how he had done it, and to his chagrin, Chris found that he had mistakenly injected the desiccant that was also in the container, not the drug. With characteristic modesty and self-effacing humor, he said that at the time he hoped this would not be a foretelling of his future in sleep research. It, of course, was not, but rather was the beginning of a remarkable career in which he made insights into a wide range of areas. In addition to the studies of the cholinergic system and human REM sleep, he contributed to understanding the phenomenon of short REM latency in depression, the switch process in bipolar disorder, sleep deprivation and light therapy as treatments for depression, and produced a landmark meta-analysis of sleep in psychiatric illness. In the process, he inspired a generation of students who now making their own contributions to sleep and circadian research.

Though Chris’s interest in sleep research grew out of his Stanford years, his attentiveness to psychiatry had begun much earlier. His father, John P. Gillin, who in the 1930s had discovered 1000-year-old Pueblo dwellings in Utah, had gone on to become a leader in cultural anthropology, with a special interest in how groups deal with mental illness. I remember Chris’s enthusiasm as he described some of his father’s studies, including ones in which he administered Rorschach tests to whole communities of indigenous peoples, and a study in which he found similarities between the techniques of the village healers and modern psychotherapists.

Another of Chris’s aspects which always struck me was his love for sports and the outdoors, which I first discovered in the St. Elizabeths years, when we played squash at the Officer’s Club at the Pentagon, and he trounced me fairly regularly. (Actually, to be clear, I should mention that beating me at squash was probably not necessarily proof of outstanding athleticism). But I remember one particularly cold Washington winter around that time, in which Chris came to the lab, describing his adventure of walking all the way across the frozen Potomac River. He later went on to climb on the Matterhorn, a story I had never heard before it came out at the festschrift in his honor in his last years. He ran in the Bay to Breakers Race in San Francisco shortly before he was found to have esophageal cancer, and in the first weeks after the diagnosis he went paragliding on the cliffs overlooking the Pacific near Scripps. In the weeks before he passed away in 2003, he went flying in a private plane.

To me, the best example of Chris’s vitality and commitment, though, happened in the late 1970s, on the day that Fred Snyder, now wheelchair-bound, returned for a visit to the NIMH lab. I was very much moved at seeing Fred’s physical frailty, and after he left, I said to Chris that the experience reminded me of the importance of taking time, amidst all our work, to smell the flowers. Chris’s response was that he could understand that, but he had the opposite reaction: it made him want to work twice as hard. I think it was that kind of drive and dedication, tempered with modesty and gentlemanly manner, that I have carried away from the years of friendship with Chris.

## Richard Jed Wyatt MD

When I first met Richard in 1971, he was not long out of training, but had already built up an impressive body of studies, many in collaboration with Fred Snyder, on the effects of drugs altering biogenic amine function on human sleep. Initially, he guided me to pursue his interests, which as I mentioned involved REM deprivation, but also the effects of tryptophan-free diets and putative endogenous sleep substances. As the years went by, he also had the wisdom to give me the freedom to move into my own areas of interest, something I tried to keep in mind later when I had my own lab.

The period in which I returned after my residency in 1975 were a watershed time for Richard in a number of ways. Chris was spending more and more time in the rapidly growing human sleep lab in Bethesda, and Richard’s own sleep program (aside from my animal work) dwindled as he focused on his ongoing schizophrenia studies. It was also at this time that he developed lymphoma, and spent some months being treated at Stanford. This and other cancers were to plague him for years to come. On his return, though, he engaged in a remarkable range of studies. He had always been interested in the possible relation of REM sleep and hallucinations, and in the process he and Chris found that schizophrenics failed to have a rebound after REM deprivation. Similarly, he looked for any possible associations between hallucinogen-induced experiences and the hallucinations of schizophrenia. He collaborated with a wide range of colleagues in studies of monoamine oxidase in platelets of schizophrenics, atrophic changes and cerebral asymmetry, effects of naloxone on psychosis, and the economic costs of schizophrenia, among many others.

As I think back about my own work with Richard, of course it’s not surprising that not all of the ideas that were floating around were successful. He had a longstanding interest in the causes of suicide and possible pathophysiologic changes associated with suicidal behavior, and he and collaborators contributed significantly to the literature. But I also recall a day in November 1978 when I spent a few anxious hours while he contemplated—and startlingly, began looking into arrangements — for sending me at once to the jungles of Guyana to explore the mass suicide, by cyanide-laced fruit juice, of Jim Jones and hundreds of his followers. When it later came out that the United States Representative Leo Ryan had just been murdered there on Jones’ orders, I was grateful that after some reconsideration this idea had not moved ahead.

On another occasion, Richard’s thoughts about REM physiology resonated with his interest in cancer therapies. He learned that some chemotherapeutic agents were more effective at higher body temperatures. Since the lack of thermoregulation during REM sleep had been well established, he suggested that we explore whether it might be possible to REM-deprive subjects at night, and then in the morning during REM rebound see if it might be easier to raise their core temperature. It was not long before we were working on the oncology unit, which had elaborate beds with heating pads, sheepskin blankets and other devices. It became clear that the contribution of prior REM deprivation was small in comparison with the massiveness of the physical methods that they employed, and the project did not go forward. Another time he thought it possible that a combination of l-tryptophan and calcium might be more effective as a sedative than either compound alone, though this turned out not to be the case.

In the early 1980s, Richard began to speculate on how an animal might develop if it grew up without REM sleep. He had previously demonstrated that the MAO inhibitor phenelzine could suppress REM in adult narcoleptic patients when given for a year. Extrapolating from human to animal, he had me administer clorgyline, another MAO inhibitor, to pregnant rats; when the pups were born, I continued to treat them until they were adults, at which time I did sleep studies. It turned out that although we had suppressed type A MAO by 99 per cent throughout their lives, they had normal amounts of REM sleep. We had, then, underestimated the nervous system’s ability to compensate and adapt. Our hope had been to publish a paper entitled ‘Growing up without REM sleep’, but we sheepishly changed it to ‘Lifetime monoamine oxidase inhibition and sleep’ [65]. And once again I experienced a sense of wonder, much as I had in the methysergide, physostigmine, and B-10 studies.

I relate these last stories to try and express both Richard’s commitment and creativity. In those years, I never knew what would be coming next. And if some ideas did not work out, many more would result in important contributions to understanding sleep and psychiatric disorders. On a more personal level, in the years before succumbing to lung cancer in June 2002 he left a legacy of dealing with illness with modesty, humor and grace, lessons from which I have tried to learn. He and Chris had been born and died within about a year of each other, passing away in their early 60s.

## Floyd E. Bloom PhD

Any account of Floyd Bloom’s accomplishments would be a daunting task, so I will mention only that during his career he was, among other things, the chairman of Neuropharmacology at the Scripps Research Institute, the president of the American Association for the Advancement of Science, and the editor of the journal *Science.* But I would like to mention only one minor incident, which took place in 1971 when he was in his mid-thirties and was director of the NIMH division located in the William A. White building at St. Elizabeths.

At the time I was a young doctor, unhappy to be pulled out of my psychiatry residency and relegated to the back wards of a huge federal hospital. I knew of Floyd because he had given guest lectures at Washington University, and I was quick to ask if I might meet him. I was happily surprised when he agreed to see me, but what then took place was even more unexpected. It had not been my experience that a director of laboratory chiefs would be likely to spend a lot of time with a young trainee, but in fact we talked for about an hour, during which he asked me about my background and my plans for research, and told me about the work he was doing.

When I asked Floyd if there might be some way I could join his group, he regretfully told me that because of the draft, all the positions were filled well in advance, with long waiting queues, but that he would see if he could work something out. A few weeks later, I got a call from him: someone in Richard Wyatt’s laboratory had unexpectedly quit, and was I by any chance interested in sleep research? Needless to say, I accepted at once, and he hired me by the creative expedient of arranging for the PHS to assign me as medical officer for the building. And so began my life studying sleep. As time went on, Floyd moved to Scripps Research Institute, where I occasionally went to meetings, and my memory was that he always had a pleasant, welcoming manner, giving me the sense of being part of the community. Later, when I had my own lab, I tried to follow his example of spending time and showing interest in fledgling scientists. I am glad that this paper gives me an opportunity to express my thanks.

## Data Availability

No new data were generated in support of thispaper, which is essentially a review article.

## Biography

**Wallace (Wally) Mendelson**, MD is Professor of Psychiatry and Clinical Pharmacology (ret) at the University of Chicago, and a Distinguished Fellow of the American Psychiatric Association. During his career he has directed the Section on Sleep Studies at the National Institute of Mental Health, the Sleep Disorders Centers at SUNY Stonybrook and the Cleveland Clinic Foundation, and the Sleep Research Laboratory at the University of Chicago. While at Stonybrook he founded one of the first ASDA/AASM accredited fellowships in sleep medicine, and subsequently established two more in Cleveland and Chicago. He was President of the Sleep Research Society in 1997. Throughout these years he maintained research programs at both basic science and clinical levels, performing the work described in this paper as well as in other areas including sleep in medical and psychiatric disorders, sleep and aging, the impact of insomnia, and the clinical use of hypnotics. In addition to professional papers, Dr. Mendelson has written 13 books on sleep and its disorders, as well as the history of psychiatry and psychopharmacology. His interest in the history of medicine is reflected in his current role as a Fellow of the Faculty of History and Philosophy of Medicine and Pharmacy at the Worshipful Society of Apothecaries, London. He is the recipient of the Academic Achievement Award from the American Sleep Disorders Association in 1999 and a special award for excellence in sleep and psychiatry from the National Sleep Foundation in 2010.
